# The impact of cannabinoids on reproductive function

**DOI:** 10.1530/REP-24-0369

**Published:** 2025-04-03

**Authors:** Reese S Cameron, Genevieve A Perono, Christian D Natale, James J Petrik, Alison C Holloway, Daniel B Hardy

**Affiliations:** ^1^ Department of Obstetrics and Gynecology, McMaster University, Hamilton, Ontario, Canada; ^2^ The Children’s Health Research Institute, The Lawson Health Research Institute, Departments of Obstetrics and Gynecology and Physiology and Pharmacology, Western University, London, Ontario, Canada; ^3^ Department of Biomedical Sciences, University of Guelph, Guelph, Canada

**Keywords:** reproductive function, ovary, placenta, sperm, cannabinoids

## Abstract

**In brief:**

This review article summarizes the effects of pre- or peri-conceptual exposure to cannabinoids on female and male reproductive function, along with pregnancy outcomes from 2014 to 2024. In particular, it addresses the gaps in knowledge regarding the specific contributions of the major constituents of cannabis, THC and CBD, on reproduction.

**Abstract:**

With increased use of cannabis worldwide, especially in our young adult population, there is a great impetus to understand the impact of cannabis and its constituents (i.e. THC and CBD) on pregnancy, fetal outcomes and male and female reproductive function. This review assessed the current evidence (2014–2024) regarding the effects of cannabinoids on reproductive function (male, female and pregnancy) in animal and human studies. In particular, pre- or periconceptual exposure to cannabinoids were assessed to determine their effects across the lifespan along with transgenerational effects. From the outcomes of this review, we conclude there is a greater need for future preclinical and clinical studies to assess how various routes of cannabinoid exposure along with differing mixtures of cannabinoid constituents may interact to impede reproductive health. Collectively, the outcomes of these studies are important to clinicians and regulatory agencies in the context of functional evidence to support policy and decision-making regarding the safety of cannabis use.

## Introduction

**Figure 1:**
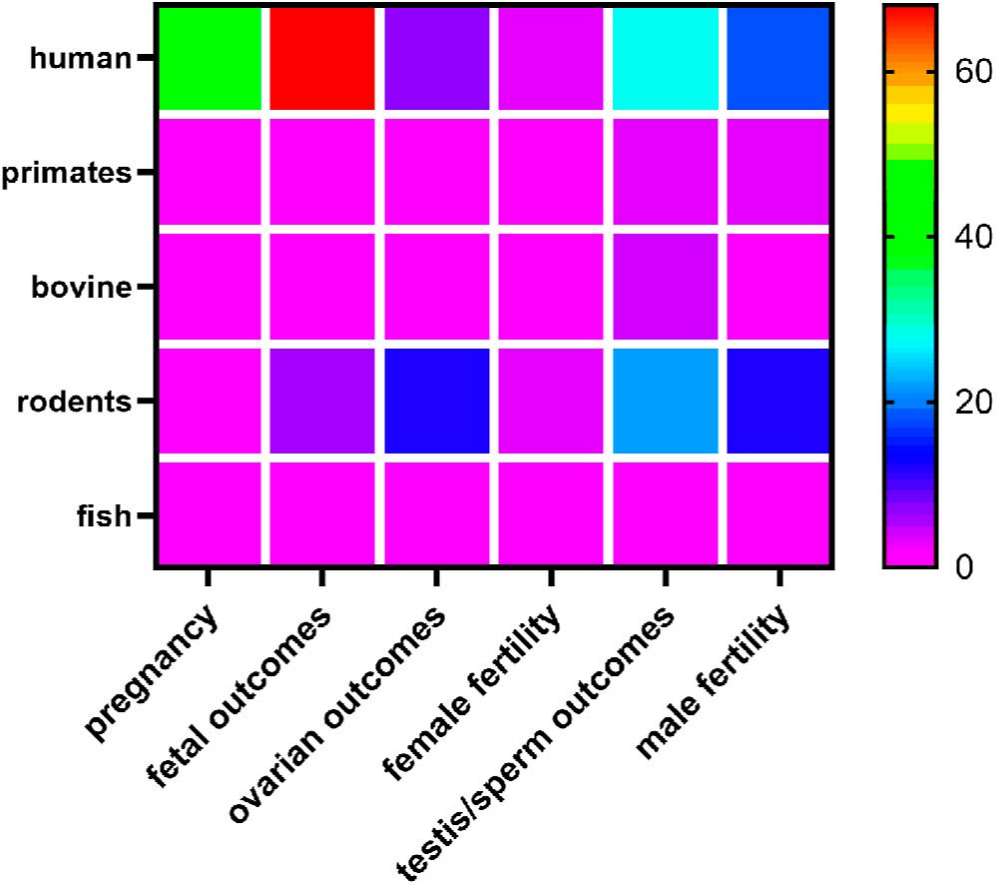
Research density heat map of areas where abundant or limited research from January 2014 to August 2024 on the effects of cannabinoids on reproductive outcomes and function. The x and y axis depict species and area of research, respectively, with density of publications depicted by colored squares. Overall, 236 studies were included. Specifically, for pregnancy/fetal outcomes, it should be noted that over 250 publications were identified in our search, but after review, only 113 were included as relevant.

The Cannabis plant contains more than one hundred distinct phytocannabinoids, including the well-known compounds delta-9-tetrahydrocannabinol (THC) and cannabidiol (CBD), along with a wide variety of terpenoids, flavonoids and alkaloids (Blebea *et al.* 2024, Laaboudi *et al.* 2024). With the increased access and popularity of cannabis over the past few decades (Degenhardt *et al.* 2013), there is a greater chance for impact on human well-being including reproductive health. With changes in legalization/decriminalization around the world, cannabis use is increasing, in particular among individuals aged 18–29 years, a period of prime reproductive potential (Imtiaz *et al.* 2023). Yet, there is limited information regarding the impact of cannabinoid exposure on reproductive health. Therefore, the goal of this review is to assess the current evidence regarding the effects of cannabinoids on reproductive function (male, female and pregnancy outcomes) in animal and human studies. Searches were conducted on PubMed to collect relevant papers from January 2014 to August 2024 based on the following keywords: ‘cannabinoids’, ‘THC’, ‘CBD’, ‘marijuana’, ‘testes’, ‘ovary’ and ‘pregnancy’. If papers identified placental but not pregnancy outcomes, they were excluded, as were papers that did not report on fetal growth, fetal loss or congenital anomalies. Although most papers utilized animal and cell models, studies that involved human participants were prioritized where possible. These searches were intended to serve as the basis for a narrative review evaluating recent findings on the effects of pre- or periconceptual exposure to cannabinoids; as such, relevant studies before 2014 were not included. As indicated in the research density heat map (Fig. 1), 236 articles were included and evaluated for this review, noting the different species involved and areas of abundant or limited research related to cannabinoid effects on reproductive outcomes.

## Pharmacokinetics of cannabinoids

Without question, the route of cannabis use (i.e. inhalation vs oral exposure) will impact the pharmacokinetics of THC and CBD *in vivo* (Grotenhermen 2003). With respect to THC, inhaled THC can peak in within 3–10 min after smoking with bioavailability in the range of 10–35% (Lindgren *et al.* 1981). However, with oral consumption, absorption is slower and more erratic, peaking 60–120 min, but in some cases, 4–6 h (Frytak *et al.* 1984, Law *et al.* 1984). The liver, through the actions of Cyp2c subfamily, can metabolize THC by first hydroxylation to 11-OH-THC, then by further oxidation to THC-COOH and finally by glucuronidation to 11-nor-9-carboxy-THC glucuronide (CO-O-glucuronide) for storage in tissues (Leighty 1973).

Inhaled CBD has a similar pharmacokinetic profile as THC, with an average bioavailability of 11–45% (Agurell *et al.* 1981, Ohlsson *et al.* 1986), whereas ingested CBD follows a similar course of absorption as THC (∼6 h) (Agurell *et al.* 1981, Consroe *et al.* 1991). Ultimately, CBD is metabolized in the liver via hydroxylation and oxidation of C-7, followed by further hydroxylation in the pentyl and propenyl groups to yield derivatives of CBD-7-oic acid (Harvey & Mechoulam 1990).

**Figure 2:**
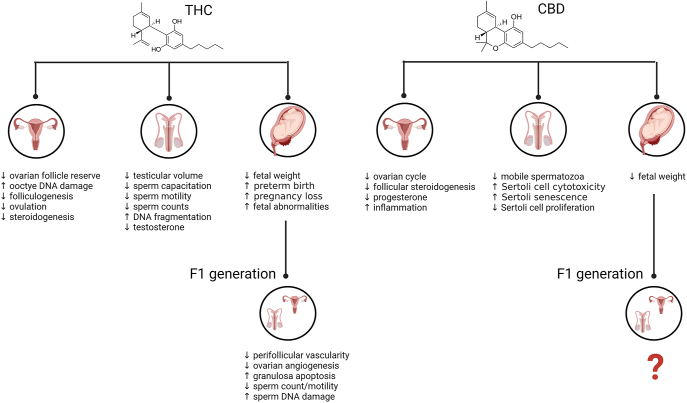
Summary of the Effects of THC and CBD on pregnancy, male and female reproductive outcomes. Created in BioRender. Hardy, D. (2025) https://BioRender.com/v38u392.

In humans and animal pregnancies, THC can rapidly cross the placenta, although the 11-OH-THC and THC-COOH metabolites cross with less efficiency (Bailey *et al.* 1987). Studies indicate that 10–28% of maternal concentrations are detected in the fetal plasma, with 2–5× higher concentrations in fetal tissues (Bailey *et al.* 1987, Hutchings *et al.* 1989). Pathology studies indicate that in pregnant cannabis users, the concentrations of THC range from ∼100 to 432 ng/g in the placenta, and from 3.9 to 281.7 ng/g in aborted fetal tissues (Falcon *et al.* 2012). While human studies on maternal CBD plasma concentrations in human pregnancy are limited, CBD has been reported in the range of 10–335 ng/g in human umbilical cord tissue from fetuses exposed to cannabis in pregnancy (Kim *et al.* 2018). While most studies have previously looked at the effects of *cannabis* and/or THC alone on reproductive outcomes, recent studies have also addressed the safety of CBD, given CBD products have skyrocketed over the past few years, and are perceived and marketed as a safe and therapeutic constituent (Soleymanpour *et al.* 2021, Goodman *et al.* 2022). The following review will address what is known about both major cannabis constituents and identify any gaps in knowledge related to CBD and reproduction. A summary of these major phenotypic effects of THC and CBD on male and reproductive function and pregnancy outcomes is found in Fig. 2.

## Implications on female reproduction

### The endocannabinoid system (ECS) in the female reproductive system

The mammalian hypothalamo–pituitary–ovarian (HPO) axis contains all of the components necessary to respond to exposure to cannabinoids. A study by El-Talatini *et al.* (2009) characterized expression of the main components of the ECS in the human ovary. CB1/CB2 receptor expression was identified in granulosa cells of primordial, primary, secondary and tertiary follicles and the corpus luteum and corpus albicans. Anandamide levels in follicular fluid were correlated with ovarian follicle size and maturity and could discriminate between immature and mature oocytes.

While the ovary contains both CB1/CB2 receptors, CB1 receptors can be found in the hypothalamus (Gammon *et al.* 2005, El-Talatini *et al.* 2009). Central nervous system regulation of the HPO axis involves the release of neurotransmitters that can impact the release of GnRH and activation of the system. As CB1 receptors are expressed on CNS neurons, cannabinoids can have an impact on GnRH secretion, which can influence ovarian function (Grant *et al.* 2018). Stimulation of CB1 receptors in the hypothalamus reduces GnRH release and mutes activation of the anterior pituitary (Gammon *et al.* 2005), which can impact release of the gonadotropic hormones. Given these outcomes, cannabinoids are generally considered to have a negative influence on ovarian function and fertility and cannabinoid exposure has been linked to both decreased folliculogenesis and ovulation in the rat (Adashi *et al.* 1983). This disruption of ovarian function has been linked to elevated risk of infertility in women trying to become pregnant (Mueller *et al.* 1990).

### Effect of endocannabinoids on granulosa cells

Granulosa cells regulate many aspects of follicular and luteal development and function. As these cells express CB1/CB2 receptors, there has been interest in identifying the impact of cannabinoid exposure on these cells. Exposure of rat granulosa cells to Δ9-THC results in significant changes in gene and hormone expression and incidence of cell death. THC exposure increased the rate of proliferation in rat granulosa cells, which was accompanied by an increase in protein expression of vascular endothelial growth factor (VEGF) and secretion of prostaglandin E2 (Martínez-Peña *et al.* 2022*a*). THC also protected granulosa cells against pro-apoptotic stimuli *in vitro* (Martínez-Peña *et al.* 2022*a*). In rat ovaries, THC administration has also resulted in decreased steroidogenesis, with reduced hydroxysteroid dehydrogenase activity in granulosa and theca compartments of the follicles (Zoller 1985). In bovine granulosa cells, exposure to THC affected DNA methylation in granulosa cells and the authors hypothesized that the implications of these epigenetic changes would be impaired oocyte maturation and fertilization potential (Floccari *et al.* 2024). Oocyte quality has been reported to be inversely associated with THC exposure, with women exposed to THC exhibited poorer-quality oocytes and lower pregnancy rates compared to women not exposed (Klonoff-Cohen *et al.* 2006). In human follicles, in which oocyte retrieval was unsuccessful, follicular fluid levels of AEA were lower, suggesting that AEA could be important in regulating folliculogenesis and oocyte maturation and quality (Schuel *et al.* 2002). Although most research on cannabinoid impacts on fertility have involved THC, there is limited research focused on the impact of CBD exposure and ovarian function. This research is becoming more relevant, given the popular use of CBD. Preclinical research on CBD in rats has also shown that CBD administration can inhibit the ovarian cycle and disrupt follicular steroidogenesis (Reich *et al.* 1982). In human granulosa cells, CBD administration increased the release of inflammatory factors such as COX2, IL6 and IL8 and interfered with progesterone synthesis (Eubler *et al.* 2023).

### Prenatal exposure to cannabinoids and ovarian function

**Figure 3:**
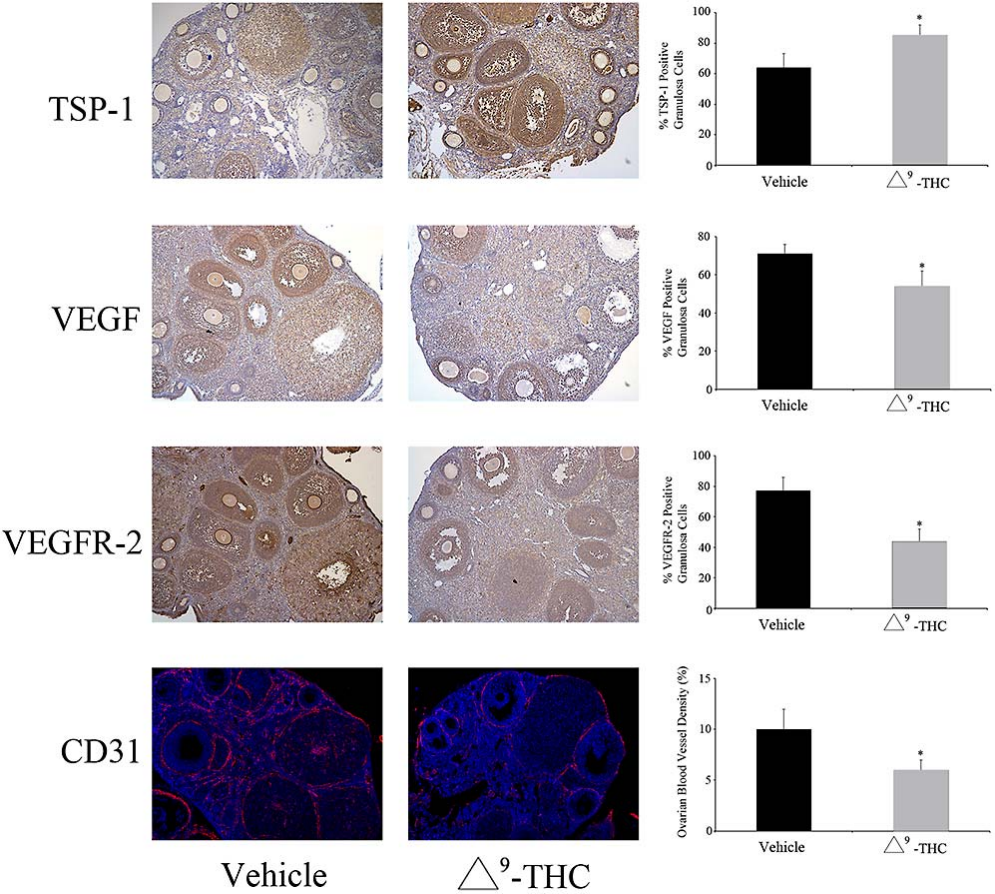
Expression of thrombospondin 1 (TSP-1), VEGF, vascular endothelial growth factor receptor 2 (VEGFR-2) and CD31 in vehicle (*n* = 5) and prenatally Δ9-THC-exposed (*n* = 6) rat ovaries. Mean ± SEM. Student’s *t*-test; *P < 0.05. Adapted with permission from Martínez-Peña AA, Lee K, Petrik JJ, *et al.* 2021 Gestational exposure to Δ9-THC impacts ovarian follicular dynamics and angiogenesis in adulthood in Wistar rats. *J Dev Origins Health Dis* **12** 865–869.

As cannabinoid exposure during pregnancy is on the rise (Young-Wolff *et al.* 2017, Corsi *et al.* 2019, Volkow *et al.* 2019), it is important to evaluate the consequences of cannabis and its constituents on fetal, postnatal and adult ovarian function. Exposure to Δ9-THC *in utero* in rats resulted in significant ovarian reprogramming in postnatal life (Martínez-Peña *et al.* 2021). Ovaries from exposed rats had reduced perifollicular vascularity, with a shift in the balance of pro- and anti-angiogenic factors, and a resultant acceleration of folliculogenesis. Similarly, studies indicate that activation of CB1/CB2 receptors during gestation altered the ovarian reserve in young adult and adult rats, suggesting that a reprogramming of follicular dynamics occurs following *in utero* exposure to cannabinoids, which can affect ovarian function in adult life (De Domenico *et al.* 2017, Castel *et al.* 2020). Furthermore, gestational JWH-133 (a CB2R agonist) exposure led to depleted primordial and primary follicles in mouse ovaries of exposed neonates attributed to a decrease in both ovarian reserve and reproductive capacity (De Domenico *et al.* 2017). Another study evaluated the long-term impact of Δ9-THC exposure *in utero* on postnatal ovarian development in rats. In this study, Δ9-THC treatment resulted in an increase in anti-angiogenic factor TSP-1 and a decrease in the expression of both VEGF and its receptor VEGFR-2; all of which were associated with a decrease in ovarian blood vessel density (e.g. CD31, Fig. 3) (Martínez-Peña *et al.* 2021). Moreover, these changes were associated with accelerated follicular development and evidence of follicular arrest (Martínez-Peña *et al.* 2021). *In utero* exposure to Δ9-THC also resulted in significant changes in several microRNAs (miRNAs), notably an increase in miR-122-5p, with subsequent downregulation of its target insulin-like growth factor 1 receptor (*Igf1r*) (Martínez-Peña *et al.* 2022*b*). This decrease in the *Igf1r* expression was associated with increased follicular granulosa cell apoptosis, further suggesting *in utero* reprogramming of postnatal ovarian function occurred during maternal THC exposure.

### Early/adolescent exposure to cannabinoids and female fertility

There has been concern that exposure to cannabinoids in adolescents can have detrimental effects on ovarian function. A study showed that peripubertal mice exposed to THC had a 50% depletion of their ovarian follicle reserve, and of the follicles that remained, there was evidence of DNA damage in the oocytes (Lim *et al.* 2023).

However, exposure to cannabinoids has also been shown to have protective effects in the ovary. In a mouse model of ovarian hyperstimulation syndrome (OHSS), treatment with CBD reduced expression of VEGF and associated vascular permeability and decreased symptoms associated with OHSS (Tahermanesh *et al.* 2022).

Another ovarian-based disorder has been associated with cannabinoid exposure. Polycystic ovarian syndrome (PCOS) is a complex multifactorial metabolic and endocrine disorder that is the leading cause of ovary-based infertility in women of reproductive age (Azziz *et al.* 2016). Women are diagnosed with PCOS when they exhibit two of the three following features: chronic anovulation, biological or clinical hyperandrogenism and polycystic ovaries. The pathophysiology of PCOS also often involves low-grade inflammation and oxidative stress, which further exacerbates ovarian function (Mancini *et al.* 2021). Exposure to Δ9-THC in rats has shown beneficial effects in PCOS, with reduced inflammation and oxidative stress and a resumption of ovarian function (Zavareh *et al.* 2024). In a preclinical animal model of PCOS, THC decreased oxidative stress, reduced inflammation and shifted the balance of M1/M2 macrophages toward a M2 phenotype (Zavareh *et al.* 2024). However, while there is evidence that cannabinoids may have a therapeutic effect in PCOS, there are also reports that activation of the ECS can have deleterious effects. Activation of ECS and overexpression of CB1/CB2 can alter glucose metabolism induce insulin resistance in women with PCOS (Juan *et al.* 2015). To date, there is limited information on the impact of CBD on PCOS, and this is an area of research that warrants further investigation.

## Implications on pregnancy outcomes

### Cannabis use during pregnancy

While some studies suggest that smoking is the most common mode of cannabis use during pregnancy (Ko *et al.* 2020), others report a widely varying range of modes of use including vaping and edibles (Mian *et al.* 2023). For those who smoke cannabis, there is exposure to cannabinoids and polycyclic aromatic hydrocarbons (including benzo[a]pyrene) resulting from combustion processes (Benevenuto *et al.* 2022). There is clear evidence that exposure to PAHs in general, and benzo[a]pyrene specifically, can affect placental development and function (Dai *et al.* 2023), suggesting that some of the association between cannabis exposure and reduced fetal growth in people who smoke cannabis may be driven by the PAH exposure. However, cell culture studies have demonstrated that THC, CBD and some of the other minor cannabinoids (cannabidivarin and cannabigerol) when administered as a single compound can adversely affect human placental trophoblast cell function *in vitro* (Lojpur *et al.* 2019, Walker *et al.* 2020, 2021, Alves *et al.* 2024, Podinic *et al.* 2024). Similarly, maternal exposure to THC and CBD individually in rats have been shown to cause impaired placental development in association with reduced fetal growth *in vivo* (Natale *et al.* 2020, Allen *et al.* 2024). Results indicating adverse pregnancy outcomes with CBD alone are particularly concerning as recent reports suggest that up to one in five pregnant individuals in North America use a cannabidiol-only containing product during pregnancy (Bhatia *et al.* 2024) despite the lack of information regarding the safety of these products.

Marijuana (*Cannabis sativa*) is one of the most widely used psychoactive substances in the world, with an estimated 228 million users in 2022 (UNODC 2024). In recent years, with increasingly liberal approaches to cannabis (e.g. legalization and decriminalization), recreational cannabis use has steadily risen among many demographic groups including pregnant individuals (Singh *et al.* 2020). Indeed, recent estimates suggest that approximately 3–7% of pregnant individual in North America use cannabis (Carlier *et al.* 2020, Singh *et al.* 2020, Hayes *et al.* 2023) in large part to treat the medical symptoms of anxiety/depression, pain, fatigue and pregnancy-related nausea and vomiting (Carlier *et al.* 2020, Besse *et al.* 2023). Many pregnant individuals have reported that they do not believe cannabis to be harmful to their baby (Weisbeck *et al.* 2021, Vanstone *et al.* 2022). However, there are now a number of human studies and animal experiments which have shown that exposure to cannabis or the major cannabinoids in cannabis (i.e. Δ9-THC and CBD) during pregnancy can result in adverse pregnancy outcomes including fetal growth restriction, preterm birth (PTB), perinatal mortality and congenital anomalies. As a result, the US Surgeon General (US Department of Health & Human Services 2019), the American College of Obstetricians and Gynecologists (ACOG 2017) and the Society of Obstetricians and Gynecologists of Canada (SOGC, (Graves *et al.* 2022)) have all issued recommendations advising against cannabis use during pregnancy. However, these agencies acknowledge that it is difficult to be certain about the specific effects of marijuana use during pregnancy due to confounding factors including tobacco use, polysubstance use and socioeconomic conditions. The goal of this particular section is to evaluate the evidence from clinical studies and animal/cell-based experiments regarding the effects of cannabis and its constituents on adverse neonatal and pregnancy outcomes.

### Impaired fetal growth

Aberrant fetal growth *in utero* resulting in babies which are low birth weight (LBW) or growth restricted (SGA: <10th percentile given sex and gestational age) is associated with increased infant morbidity and an increased risk of chronic disease across the life course (Pels *et al.* 2020, Nüsken *et al.* 2024, Oulerich & Sferruzzi-Perri 2024). There is now strong evidence from systematic reviews of human studies suggesting an association between cannabis use during pregnancy and impaired fetal growth (Baía & Domingues 2022, Lo *et al.* 2024). Baía & Domingues (2022) reported that pregnant individuals who use cannabis during pregnancy are at increased risk for LBW (OR = 1.52; 95% CI: 1.18–1.96) and SGA (21 OR = 1.47; 95% CI: 1.38–1.58). Similarly, Lo *et al.* conducted a systematic review and meta-analysis to determine if prenatal cannabis use increased the likelihood of SGA and reported that in the unadjusted analysis of 21 studies with 4,582,445 individuals, there was an increased risk of SGA (OR = 2.06; 95% CI: 1.76–2.41) (Lo *et al.* 2024). Importantly, Lo *et al.* also adjusted for prenatal tobacco use, a critically important consideration, as maternal smoking is also associated with an increased risk of impaired fetal growth (Bouquet *et al.* 2023, Arabzadeh *et al.* 2024). The increased risk of SGA with cannabis use persisted after adjustment for several confounders, including tobacco use (aOR = 1.76; 95% CI: 1.39–1.62) (Lo *et al.* 2024). The mechanisms by which cannabis exposure can result in impaired fetal growth are as of yet not fully understood but likely involve impaired placental function (Metz *et al.* 2023).

### Preterm birth

Recent systematic reviews have reported an increased risk of preterm birth (<37 weeks gestation) with cannabis use. In evaluating 31 studies with over 14 million participants, Lo *et al.* reported an unadjusted OR of 1.62 (95% CI: 1.43–1.83) for preterm birth, whereas Baia and Domingues report an unadjusted OR of 1.39 (95% CI: 1.28–1.51). This relationship between PTB and cannabis use is higher in studies that did not adjust for prenatal tobacco exposure (Duko *et al.* 2023) but still persists when adjusted for confounding including maternal smoking (Duko *et al.* 2023, Lo *et al.* 2024). As exposure to PAHs such as benzo[a]pyrene have been reported to reduce gestation length in rodents (Laknaur *et al.* 2016) and humans (Cathey *et al.* 2024), it is plausible to hypothesize that PAHs in cannabis smoke might be the principal driver of the association between cannabis use during pregnancy and PTB. However, as THC has been shown to affect glucocorticoid (Natale *et al.* 2020), prostaglandin (Martínez-Peña *et al.* 2022*b*) and endocannabinoid signaling pathways in rats (Martínez-Peña *et al.* 2021), all of which have been implicated in the timing of human parturition (Kozakiewicz *et al.* 2021, Li *et al.* 2021, Karampitsakos *et al.* 2024), there is also the possibility that THC and other constituents of cannabis might also affect key molecular pathways underlying the timing of parturition, despite there being no evidence of any significant change in gestation length in animal studies where rodents were exposed to THC alone (Breit *et al.* 2020, Natale *et al.* 2020).

### Pregnancy loss, fetal/neonatal demise

In their systematic review of neonatal outcomes with cannabis use in pregnancy, (Lo *et al.* 2024) reported an increased risk of perinatal mortality in association between prenatal cannabis use (OR = 1.84; 95% CI: 1.47–2.20). However, they noted that in the studies included in their analysis that perinatal mortality could be defined as stillbirth, miscarriage/spontaneous abortion or death before hospital discharge (i.e. neonatal death). Similarly, rats exposed to cannabis smoke starting in early gestation (i.e. starting at gestational day 6; term = 21–23 days) had increased rates of spontaneous abortion and fetal resorption (Akingbade *et al.* 2021). Importantly, fetal loss appears to be specific to animal models of smoke exposure as exposure to physiological concentrations THC and CBD via *i.p.* injections did not result in a reduction in litter size or the number of resorptions in rats (Natale *et al.* 2020, Allen *et al.* 2024). Taken together, these data suggest that it may be the PAHs in cannabis smoke which contribute to pregnancy loss. This is consistent with literature suggesting that smoking and PAH exposure are both associated with an increased risk of pregnancy loss (Dai *et al.* 2023, Tong *et al.* 2024).

### Congenital anomalies

There are two recent systematic reviews and meta-analyses which have explored the association between prenatal exposure to cannabis and major structural birth defects (Delker *et al.* 2023, Tadesse *et al.* 2024). Delker *et al.* reported a pooled adjusted odds ratio of 1.22 (95% CI: 1.00–1.50) for any congenital anomaly, whereas, Tadesse *et al.* reported an increased risk of any birth defect (OR = 1.25, 95% CI: 1.12–1.41) and specifically an increased risk of cardiovascular/heart, gastrointestinal, CNS and genitourinary systems defects (Tadesse *et al.* 2024). While there is a paucity of animal studies which have explored the effects of cannabis or its constituents on structural defects, the studies that do exist have reported that prenatal exposure to THC and CBD can cause structural (Lee *et al.* 2021, 2024, Le *et al.* 2024, Robinson *et al.* 2024) and functional (Lee *et al.* 2021, 2024) cardiac deficits in rhesus macaques and rat offspring, suggesting that exposure to cannabis and its constituents independently of route of administration may increase the risk of birth defects.

## Implications on male reproduction

### The ECS in the male reproductive system

Similar to the female reproductive system, the male reproductive system (i.e. testis, seminal vesicles and spermatozoa) exhibits all the components of the ECS, including both cannabinoid receptors (CB1R/CB2R and GPR55), the synthesizing enzymes (NAPE-PLD and DAGL) and the degrading enzymes (FAAH and MAGL) (Lewis *et al.* 2012, Ückert *et al.* 2018, Nielsen *et al.* 2019). In the fetal life, the expression of *Cb1r* increases in the mouse testis with development, while both *Cb1r/Cb2r* increases in the developing ovary (De Domenico *et al.* 2017). Other studies have demonstrated that the endocannabinoids AEA and 2-AG are also present in the human testis by fetal life, and can be altered *ex vivo* by exposure to THC or CBD (Dochez-Arnault *et al.* 2023). The impact of the endocannabinoids on the male reproductive system are still being extensively investigated, but in a cohort of 200 young Swiss men, seminal fluid AEA concentrations were inversely correlated to sperm motility, with THC metabolites linked to lower concentrations of endocannabinoids (Zufferey *et al.* 2020). In addition, in healthy fertile men, mature spermatozoa has also been associated with higher levels of CB1R and CB2R, with CB1R significantly related to variations in sperm morphology and CB2R related to both sperm morphology and linearity index (Hazem *et al.* 2020).

A greater understanding of the importance of the ECS in the male reproductive system has emerged from cell culture and preclinical studies using agonists/antagonists of CB1R/CB2R. Paternal exposure of the CB2R agonist, JWH-133, in CD-1 rats has been demonstrated to lead to seminiferous tubule degeneration, partial germ cell depletion, disorganized seminiferous epithelium concomitant with decreased sperm concentrations, sperm motility and testosterone levels (Mutluay *et al.* 2022). Furthermore, in immature CD1 mice, agonists (JWH-133) and antagonists (AM630) of CBR2 disrupted the temporal dynamics of spermatogenesis through changes in posttranslational histone modifications (Di Giacomo *et al.* 2016). However, it is noteworthy that the testis and spermatogenesis did recover after 45 days. Interestingly, in mouse testicular explants (PND6.5), long-term exposure to JWH-133 led to enriched haploid germ cells and an increase in post-meiotic germ cells density, suggesting CBR2 improves *in vitro* entry into meiosis and differentiation of spermatogonia (Dumont *et al.* 2021). Furthermore, JWH-133 increases meiosis in fetal (GD13.5-GD15.5) male gonads from CD-1 mice (De Domenico *et al.* 2017). Looking at the transgenerational effects of CB2R activation, elegant studies by Innocenzi *et al.* demonstrated that paternal exposure of JWH-133 to 5 week male mice led not only to decreased numbers of spermatozoa and sperm motility, but also to reduced placental area and fetal growth restriction (Innocenzi *et al.* 2019). This impairment in fetal growth is attributed to alterations in DNA methylation and hydroxymethylation of imprinted genes in the JWH-133 exposed sperm which are conserved in the placenta. In contrast, gestational exposure to JWH-133 led to fetal growth restriction in male and female mice offspring, but no germ cell defects were apparent in male offspring (De Domenico *et al.* 2017).

### Effects of THC and CBD on the spermatogenesis and testicular outcomes

Given the increased popularity of cannabis in users of reproductive age, it is imperative to define the effects of cannabis and/or the major cannabinoid constituents, THC and CBD, on male reproductive development and spermatogenesis, along with potential transgenerational effects. Collectively, studies in rhesus macaques indicate that THC can decrease testicular volume (via increased atrophy), testosterone and male fertility, but this could be reversed with cessation of THC (Hedges *et al.* 2022, 2023). With respect to sperm quality, studies in several animal species implicate that THC, via various routes of exposure, impacts sperm capacitation and can decrease sperm motility, sperm kinematics and sperm counts (Mobisson *et al.* 2022, Shi *et al.* 2022, van Losenoord *et al.* 2022, Truong *et al.* 2023). Mechanistically, while cannabis/THC has been demonstrated to alter DNA methylation in humans and rat sperm in a similar manner (Murphy *et al.* 2018), the effects on human sperm function are more contradictory. Numerous human studies indicate that exposure to cannabis can lead to higher percentage of abnormal sperm, chromosomal abnormalities and DNA fragmentation, along with impaired testosterone and decreased sperm count (Gundersen *et al.* 2015, Verhaeghe *et al.* 2020, Hehemann *et al.* 2021, Belladelli *et al.* 2023). However a systematic review and several other reports would indicate there is no correlation between cannabis use and sperm quality(Nassan *et al.* 2019, Belladelli *et al.* 2021, 2023, da Silva *et al.* 2023, Khan *et al.* 2023). While further large-scale studies are necessary, it is clear that stratifying outcomes by subfertility/endocrine profiles, ethnicity, duration of use and ratio of CBD/THC in the cannabis plant is necessary. This is especially pertinent to the duration of use, given studies in humans and rhesus macaques clearly indicate that cessation of cannabis use can improve endocrine profiles, male fertility and reduce the number of sperm exhibiting epigenetic alterations in male development (Schrott *et al.* 2021, Hedges *et al.* 2023).

Despite being marketed as safe, recent studies demonstrate that CBD may be detrimental to male reproductive health and function. In primary human Sertoli cells and the mouse TM4 Sertoli cell line, CBD led to concentration and time-dependent cytotoxicity through alterations in the cell cycle and increased markers of senescence (Li *et al.* 2022, 2023*a*). Transcriptomic analysis further confirmed these major effects on the cell cycle (Li *et al.* 2023*b*). The main CBD metabolites, 7-carboxy-CBD and 7-hydroxy-CBD, also inhibited Sertoli cell proliferation (Li *et al.* 2022). Moreover, in human Leydig cells, CBD and its metabolites also promoted apoptosis (Li *et al.* 2023*a*). Preclinical models in rodents further demonstrated that CBD can reduce the percentage of mobile spermatozoa and other measures of sperm quality (i.e. higher number of abnormal acrosomes) despite no effects on testis weight, sperm count or hormone levels (Carvalho *et al.* 2022). In studies with a prepubertal exposure to cannabichromene (CBC), the cannabinoid with better binding affinity, CBC exposed males also exhibited decreased spermatogenesis and steroidogenesis (Taiwo *et al.* 2023). However, to date, there is a great paucity of clinical studies regarding the effects of CBD alone on human sperm quality. One study in healthy Swiss men would suggest that cannabis users with confirmed levels of plasma CBD exhibited higher AEA, androgens and estradiol, but the contributing effect of CBD alone on semen quality could not be definitely assessed (Zufferey *et al.* 2024).

### Effects of THC and CBD on male reproductive outcomes

While there is a large amount of literature investigating the effects of endocannabinoids and/or phytocannabinoids on sperm quality, several epidemiological studies have also addressed how chronic cannabis use could impact male reproductive outcomes. A large 10-year study looking at 7,809 males indicates that cannabis users exhibited higher serum testosterone, higher Sexual Health Inventory for Men (SHIM) scores (Barbonetti *et al.* 2024) and greater sexual frequency(Shiff *et al.* 2021). However in infertile men, cannabis use led to lower testosterone levels and abnormal sperm morphology (Verhaeghe *et al.* 2020, Belladelli *et al.* 2022). Another study indicates that while cannabis use is linked to increased sexual function, the clinical significance of this is likely limited due to selection bias (Bhambhvani *et al.* 2020). When looking more closely at psychosexual function, it has been demonstrated that cannabis use in males is associated with lower sexual desire, intercourse satisfaction, higher sexual depression, lower sexual esteem and impaired erectile function (Asal *et al.* 2024). However in infertile men, mild cannabis use led to better lipid profiles and penile arterial vascular response (Barbonetti *et al.* 2024). Once again, this would imply that there are contrasting effects of cannabinoids on reproductive effects in fertile vs infertile men. While there are no clinical studies examining the effects of CBD alone on reproductive function, a preclinical study in Swiss mice demonstrated that chronic CBD exposure promotes functional impairment of the reproductive system, as indicated by delayed mounting and intromission, and reduced ejaculations (Carvalho *et al.* 2018). If exposed too early in life, there is also a chance that cannabinoids could also impact the onset of puberty. A great example of this stems from a case involving a 2-year-old boy who used cannabidiol for pediatric epilepsy. This resulted in the development of precocious puberty, thought to occur, in part, to cannabinoid-induced activation of the hypothalamic–pituitary–gonadal axis (Krishnan *et al.* 2021).

### Transgenerational effects of paternal THC or CBD exposure

While we have earlier described the transgenerational effects of CB2R activation on impaired placental development and fetal growth restriction (Innocenzi *et al.* 2019), a few studies have looked at the potential for transgenerational effects of phytocannabinoid exposure, especially with respect to CBD. Fertilization of bovine oocytes with THC-treated sperm did not impact developmental rates, but did result in blastocysts with fewer trophoblasts (Kuzma-Hunt *et al.* 2024). Cannabis vapor-exposed male mice exhibited decreased sperm count and/or motility in both F0 and F1 males, associated with increased DNA damage (Shi *et al.* 2022). Elegant studies by Schrott *et al.* indicate that paternal cannabis exposure in rats leads to differential sperm DNA methylation, which can be reduced with cannabis abstinence during late spermatogenesis (Schrott *et al.* 2022). The timing of cannabis exposure also differentially alters the DNA methylation patterns in the F1 sperm, hippocampus and nucleus accumbens (Schrott *et al.* 2022). However, regardless of the timing of cannabis extract, F1 cannabis-exposed rat hearts were increased at birth, indicating that not all paternal THC-induced developmental effects in the F1 generation can be reversed by late abstinence of use. The route of paternal cannabinoid exposure may also play a role given oral, but not *i.p*. exposure to THC led to sperm DNA methylation and transgenerational effects (Acharya *et al.* 2020).

## Conclusions and the focus of future studies

People are accessing cannabinoid products at unprecedented levels and in a variety of different forms. There is a general assumption that cannabinoid exposure is safer than both tobacco smoking and taking other drugs. As such, this has led to widespread use among young people and in our pregnant population. In addition, cannabinoid products have a growing perception of offering health benefits such as pain relief, reduced inflammation and more recently as anti-cancer strategies. With these beliefs, more people are taking cannabinoids that have comorbidities and/or other forms of sub-optimal health. Some of these health issues include reproductive disorders. In most cases, the implications of cannabinoid exposure on reproductive tissues and function are poorly understood. The interactions between cannabinoid exposure and reproductive health are complex and there is no definitive view on whether cannabinoids have a positive or negative implication. However, the research included in this review does highlight that exposure to cannabinoids has significant and sometimes dramatic impacts on reproductive tissues across the life spectrum including fetuses, neonates and young and adult people. Moreover, there is now further evidence that this can have transgenerational effects on reproductive and metabolic organ development as well. With this, it is imperative to further our understanding of the specific involvement of different cannabinoid constituents (i.e. alone vs as complex in the cannabis plant) upon the reproductive health in various populations over different stages (i.e. prepuberty vs postpuberty) of development. Furthermore, with the increasing popularity of edibles and vaping, future studies are needed to assess how these routes of cannabinoid exposure differentially affect reproductive outcomes as well. As highlighted throughout the review, there is still a great gap in knowledge with respect to how exposure to phytocannabinoids may alter the endogenous ECS to impact reproductive function. Finally, this review also implicates that despite being marketed as safe, future clinical and preclinical studies are warranted to fully investigate the impact of CBD on reproductive function and health across generations.

## Declaration of interest

The authors declare that there is no conflict of interest that could be perceived as prejudicing the impartiality of the work reported.

## Funding

This work was supported by CIHR Project Grants R4228A28 to DBH, 461152 to JJP and PJT-155981 to ACH.

## Author contribution statement

The major concepts of the review were designed by DBH, JJP and ACH, while the manuscript was written equally by DBH, JJP and ACH. RC, GAP and CDN employed the search parameters and collated the outcomes, while RC generated the research density heat map.
